# Comprehensive *in silico* Characterization of Universal Stress Proteins in Rice (*Oryza sativa* L.) With Insight Into Their Stress-Specific Transcriptional Modulation

**DOI:** 10.3389/fpls.2021.712607

**Published:** 2021-07-28

**Authors:** Shatil Arabia, Asif Ahmed Sami, Saima Akhter, Rakha Hari Sarker, Tahmina Islam

**Affiliations:** Plant Breeding and Biotechnology Laboratory, Department of Botany, University of Dhaka, Dhaka, Bangladesh

**Keywords:** universal stress proteins, rice, transcript alteration, abiotic stress, functional validation

## Abstract

In a world where climate change is real and its consequences are unprecedented, understanding of the plant adaptive capacity and native stress-responsive machinery is crucial. In recent years, universal stress proteins (USPs) have received much attention in the field of plant science due to their stress-specific transcriptional regulation. This study focuses on the extensive characterization of the *USP* gene family members in the monocot crop rice (*Oryza sativa* L. var. *japonica*). Here, we report a total of 44 *USP* genes in the rice genome. *In silico* characterization of these genes showed that domain architecture played a major role in the functional diversification of the *USP* gene family which holds for all plant *USPs*. On top of that, a higher conservation of *OsUSP* members has been exhibited with a monocot genome (*Zea mays* L.) as compared to a dicot genome (*Arabidopsis thaliana* L.). Expression profiling of the identified genes led to the discovery of multiple *OsUSP* genes that showed pronounced transcript alteration under various abiotic stress conditions, indicating their potential role as multi-functional stress-specific modules. Furthermore, expression validation of *OsUSP* genes using qRT-PCR provided a strong evidence for the utility *OsUSP* genes in building multi-stress tolerant plants. Altogether, this study provides leads to suitable *USP* candidates that could be targeted for plant breeding and genetic engineering experiments to develop stress resilient crop species.

## Introduction

Alteration of the growth environment from optimum to adverse conditions is a common occurrence in a plant’s life cycle. Being sessile, plants must exhibit a dynamic response to cope with subtle to drastic changes in their nearby regimes. Such changes can range from diverse ecological parameters such as temperature, water and nutrient availability, salt content, etc. to intrinsic factors such as accumulation of reactive oxygen species (ROS). Under such unfavorable conditions, plants can suffer numerous repercussions, e.g., arrested growth and development, lower photosynthetic capacity, abnormalities in flowering time, reduced fertility and germination rate, abated total yield, etc. (Munns, 2002; Barnabás et al., 2008). To overcome this, plants have evolved highly complex yet coordinated responses through temporal and spatial regulation of genes that can mitigate unwanted effects resulting from various abiotic stressors. Generally, perception of stressors triggers many downstream signaling cascades within plant cells, such as protein kinases, phosphatases, transcription factors, molecular chaperones and defense-related proteins that mediate a suitable response to ensure plant survival (Jung et al., 2015). However, in a world where climate change is experiencing a rapid spike, plants native stress-responsive machinery is not able to protect the cellular system from stress inflicted damages (Lesk et al., 2016). These circumstances have accelerated the search for stress-induced genetic components that hold the capacity to endow plants with enhanced stress resilience through modern biotechnological tools. In this regard, the most suitable candidates include those genes that can mediate protection against multiple stressors simultaneously. One such group of proteins that have recently come into the spotlight for their stress-specific roles in plants, are the universal stress protein (USP) family (Chi et al., 2019).

The first USP was reported in *E. coli* where the candidate protein responded to a multitude of environmental perturbations including nutrient starvation, temperature shock, oxidative and osmotic stress, heavy metal toxicity, antibiotics, etc. (Nyström and Neidhardt, 1992; Zarembinski et al., 1998; Sousa and McKay, 2001). Later, USPs have been reported in many other organisms including other bacteria, archaea, fungi, and even plants (Vollmer and Bark, 2018). Although *E. coli* USPs by structure only contain the USP-domain, plant USPs have largely diversified in function by accumulating additional accessory domains that allow them to participate in numerous varying functions (Chi et al., 2019). Consequently, the multi-functional nature of USPs is derived from their structural diversity. Information of the evolution and function of plant USPs is very limited to date. A few genes have been studied in rice, *Arabidopsis*, cotton, etc. which were found to play a key role in stress regulation (Sauter, 2002; Merkouropoulos and Tsaftaris, 2013; Jung et al., 2015). Nevertheless, these studies strongly hint to the importance of plant USPs for further studies. *OsUSP1* from rice was the first *USP* gene identified in the plant kingdom that has been involved in the activation of signaling cascade in response to ethylene during hypoxia (Sauter, 2002). It was reported that stomatal closure is enhanced by *SpUSP* during drought stress to limit the detriment in tomatoes (Loukehaich et al., 2012). Two *Arabidopsis thaliana USP* isoforms (At3g62550 and At3g53990) were reported to show enhanced expression under drought (Shinozaki and Yamaguchi-Shinozaki, 2007). Recent studies have also highlighted the role of USPs in conferring benefits during other abiotic stresses (Udawat et al., 2016). For instance, the *AtUSP* (At3g53390) gene is significantly induced by salt, osmotic stress, and wounding (Isokpehi et al., 2011). Overexpressing *AtUSP* (At3g53990) conferred tolerance against oxidative and heat stresses whereas sensitivity to these stressors was observed in *atusp* (SALK_146059) mutant lines (Jung et al., 2015). *AtUSP* was also found to be involved in cold stress (Melencion et al., 2017). Apart from that, *USP* regulates ethylene-mediated signaling and thereby modulates fruit ripening (Isokpehi et al., 2011). A tomato USP protein, SlRd2 interacts with calcineurin B-like interacting protein kinase (SlCipk6) and is involved in the regulation of ROS production (Gutiérrez-Beltrán et al., 2017). In cotton, two closely related genes *GhUSP1* and *GhUSP2*, within the USP family were reported to be drought-responsive (Maqbool et al., 2009). Moreover, *AtUSP* promoter has shown to upregulate *GUS* expression in response to abscisic acid (ABA), 1-aminocyclopropane-1-carboxylic acid (ACC), dehydration, heat, cold, salt, and osmotic stress (Bhuria et al., 2016). This is a clear indication of the functional capacity of plant USPs under multi-stress conditions. In line with such importance, forty-one *USP* genes were identified in the model plant *Arabidopsis thaliana* (Bhuria et al., 2019). Out of these, *AtUSP9* and *AtUSP12* were identified as multiple stress-responsive.

Rice is an important crop and an ideal model for the comparative study of gene functions owing to high efficiency of genetic transformation and ease of cultivation and propagation (Basso et al., 2020). With the release of the whole genome sequence of rice (Song et al., 2019), it is very convenient to comprehensively analyze an entire gene family. Recently, 38 *USP* genes were reported in rice (Japonica variety) (Chi et al., 2019). However, a detailed analysis of the functional annotation and mode of regulation of these genes is still lacking. Due to the multi-stress responsive nature of plant USPs, it is imperative to look for the suitable candidates that could be targeted for improving crop resilience under multiple environmental adversities. Here, considering the important role of USP proteins in plant stress regulation and the lack of information about this gene family in a crop species, our study aimed to screen the *USP* gene family in rice. This study provides an in-depth bioinformatics characterization of the identified *USP* genes along with their developmental and stress-specific transcriptional regulation. Overall, this study provides suitable *USP* genes for future biotechnological applications aiming to generate of multi-stress tolerant agricultural crops and serves as a framework for investigating the *USP* gene family in other important plant species.

## Materials and Methods

### Identification, Gene Nomenclature and *in silico* Analysis of *USP* Genes in Rice

To identify the *USP* genes in rice, blastp and Hidden Markov Model (HMM) search had been done. Rice [*Oryza sativa Japonica* Group (Japanese rice) cv. Nipponbare] USP protein sequences were retrieved from the publicly available genome database in the Rice Annotation Project Database (RAP-DB^1^) (Sakai et al., 2013) using *Arabidopsis USP* (At1g68300) as a query sequence. Subsequently, each of the newly identified sequences were used as a query sequence to find the other new members. To confirm the result from HMM and blastp search, the putative USP sequences were also analyzed against the Pfam database^2^ (Mistry et al., 2020). Nomenclature of rice *USPs* was given according to the chromosomal location in descending order (Hasan et al., 2021). Information on the gene locus ID, protein size and full-length cDNA, the molecular weight, theoretical pI, the number of amino acids were obtained from the RAP-DB database. The subcellular localization of the proteins was predicted using CELLO v.2.5: subCELlular LOcalization predictor^3^ (Yu et al., 2006) and WoLF pSORT^4^ software (Horton et al., 2007). ChloroP^5^ (Emanuelsson et al., 1999) was used to verify chloroplast localization.

### Exon–Intron Distribution of *OsUSP* Genes, Identification of Conserved Motifs and Amino Acid Content in OsUSP Proteins

The GFF3 annotation file for the rice genome was downloaded from the Ensembl Plants^6^ (Howe et al., 2020). Information regarding the position of exon–introns and UTR regions of rice *USP* genes were extracted from this file. For the identification of conserved motifs, MEME SUITE software (v.5.3.3) was used (Bailey et al., 2009) with all parameters were kept in default settings except the number of motifs to find was set to 10. The “Gene Structure View” option of TBtools (v.1.092) (Chen et al., 2020) was used to visualize the exon–intron distribution and conserved motifs of rice USP members. Both the modified GFF3 file and MEME SUITE XML output file was used for this task. Amino acid content was calculated using the Biopython (v.1.78) package.

### Chromosomal Localization of *OsUSP* Genes and Their Synteny Analysis With *Arabidopsis* and Maize Genome

Chromosomal distribution of each *OsUSP* gene was retrieved from the Oryzabase database for rice^7^. For the duplication study within the rice genome, data including the synonymous rate (Ks) and non-synonymous rate (Ka) values were retrieved from the plant genome duplication database^8^ (Lee et al., 2012). Duplication type and selection pressure was calculated according to Hasan et al. (2021). The estimated date (Mya, million years ago) of each duplication event was calculated by using *T* = Ks/2λ where *T* is divergence time, Ks is the number of synonymous substitutions per site, and λ is the fixed substitution rate of 6.5 × 10^–9^ mutations per synonymous site per year in grasses (Gaut et al., 1996). Syntenic relationships between rice, *Arabidopsis* and maize *USP* genes were constructed and visualized using MCScanX (Wang et al., 2012) and TBtools (v.1.092) (Chen et al., 2020), respectively. In TBtools, the “Advanced Circos” option was used for visualizing the syntenic blocks.

### Phylogenetic Relationship Between Rice, *Arabidopsis*, and *E. coli* USP Proteins Along With Their Domain Architecture

To get an idea about the evolutionary history of the rice *USP* gene family, we performed a phylogenetic analysis using the USP protein sequences of rice, *Arabidopsis*, and *E. coli*. *Arabidopsis* and *E. coli* USP protein sequences were retrieved from TAIR (Berardini et al., 2015) and UniProt (UniProt Consortium, 2021) databases, respectively. The protein sequences were aligned using the online version of MAFFT tool v.7 (Katoh et al., 2019) with default parameters. Next, gaps in the aligned regions were trimmed using the Phyutility tool (v.2.7.1) (Smith and Dunn, 2008) to retain 75% of the consensus alignment. With the trimmed multiple sequence alignment file, a phylogenetic tree was constructed using IQ-Tree v.2.1.2 (Minh et al., 2020) with 1000 ultrafast bootstrap replicates. Finally, the tree was visualized and edited using iTOL (v.6) (Letunic and Bork, 2019). The domain features were uploaded as an additional dataset in the required format (details on the iTOL help page). Domain information of each USP protein was downloaded from Pfam (Finn et al., 2014).

### Identification of Microsatellite Markers, Glycosylation and Phosphorylation Sites in OsUSPs

For the identification of SSR markers in *OsUSP* members, a microsatellite identification tool (MISA^9^) (Beier et al., 2017) was used. The minimum repeat unit was defined as 10 units for mononucleotide repeats and 5 units for dinucleotide, trinucleotide, tetranucleotide, pentanucleotide, and hexanucleotide repeats. The maximum length of sequence between two markers was set to 100 units. The number of glycosylation sites in OsUSP proteins was predicted using NetNGlyc 1.0 server^10^ (Gupta and Brunak, 2002) with a threshold of 0.5. The predicted phosphorylation sites of all OsUSP members were identified using NetPhos3.1^11^ (Blom et al., 1999, 2004) with the threshold of 0.75. Predictions were performed with all three residues (Tyr, Thr, Ser) for both generic and kinase-specific phosphorylation.

### Expression Analysis of *OsUSP* Genes at Different Tissues, Developmental Stages and Under Stress Conditions Using Publicly Available mRNA-Seq Data

Temporal and spatial expression profiles of *OsUSP* genes were retrieved from Genevestigator^12^ (Hruz et al., 2008) for different developmental stages, anatomical tissues and in response to stress conditions (Supplementary Tables 1–3). Heatmaps were generated for each type of expression data using the “Heatmap Illustrator” option in TBtools v.1.092 (Chen et al., 2020). Heatmaps of both tissue and developmental expression patterns were created using the log_10_ transformed mean expression values. As for the expression under stress conditions, the log_2_ fold change in expression data was used to generate the Heatmap.

### Analysis of *Cis*-Regulatory Elements and GO Enrichment of *OsUSP* Genes

To identify the *cis*-regulatory elements present in the putative promoter region, 1 kb upstream sequence of each *OsUSP* gene was used. Subsequently, the sequences were uploaded to the PlantCARE database^13^ (Lescot, 2002) to detect the presence of various *cis*-elements. Later, the presence of these *cis*-regulatory elements was visualized using a stacked bar plot. Gene Ontology enrichment analysis of *OsUSP* genes was performed with the help of agriGO database v.2 (Tian et al., 2017). The results were visualized in the R programming platform.

### Plant Growth and Stress Treatments

For expression profiling, a uniformly developed rice seeds of BRRI-53 variety were placed in a greenhouse (16 h photoperiod, 28 ± 2°C temperature) (Islam et al., 2015). The 15 days old seedlings were then taken for various experimental treatment such as salt (200 mM), drought (mannitol; 150 mM), oxidative (30% H_2_O_2_), cold (4°C), heat (42°C), and dehydration (air-dried), ABA (10 mM), gibberellic acid (GA3, 1 mM), salicylic acid (SA, 2 mM) (Islam et al., 2015). The untreated seedlings were considered as a control to the treated seedlings. After 16 h treatment, leaves were collected (three biological replicates), immediately frozen in liquid nitrogen, and stored at −80°C.

### RNA Isolation and RT-PCR Analysis

Rice seeds of BRRI-53 variety were grown in the controlled environmental condition of 28 ± 2°C temperature and 16 h photoperiod in a growth chamber. Fifteen days’ old seedlings were then used for various abiotic stress treatment such as salt (200 mM NaCl), drought (150 mM Mannitol), oxidative (30% H_2_O_2_), cold (4°C), heat (42°C), dehydration (air-dried) and hormonal treatment such as, ABA, GA3, and SA (Islam et al., 2015), for 16 h. The untreated seedlings were considered as a control to all these stresses. The tissues were harvested, and total plant RNA was isolated from rice shoots using TRIzol reagent (Invitrogen, United States) as per the manufacturer’s protocol (Supplementary Figure 1).

First-strand cDNA was synthesized with reverse transcriptase (Thermo Fisher Scientific, United States) by following the manufacturer’s protocol. A reaction mix (12 μl) was prepared in a PCR tube containing 10 μl of DNase treated RNA, 1 μl of DEPC-H_2_O and 1 μl oligo dT primer. The reaction mixture was incubated at 65°C for 5 min and chilled down on ice immediately for 5 min. Then, a master mix of 4 μl containing 5X reaction buffer, 1 μl of Riboblock RNAse inhibitor, 1 μl of Revertaid and 2 μl of 10 mM dNTPs mix was added in the tubes. The mixture was incubated at 42°C for 60 min. After the first strand synthesis, the reaction was terminated by heat inactivation at 70°C for 5 min. The expression of individual genes was measured with gene-specific primers by real-time PCR analysis with a cycler Applied biosystem 7500 and SYBR Green mixture (Bio-Rad, United States) (Supplementary Table 4). The relative expression of specific genes was quantitated with the 2-ΔΔCt calculation method (Livak and Schmittgen, 2001), where ΔΔCt was the difference in the threshold cycles and the reference gene, which was rice *eEF1* for the expression analyses (Islam et al., 2015). The sequences of gene-specific primers are provided in Supplementary Table 5.

### Statistical Analysis

Statistical analysis was performed for the relative normalized expression data from three biological replicates under each treatment (*n* = 3). The paired student’s *t*-test was performed for each treatment against the respective controls to determine the significance level that were marked with ^∗^, ^∗∗^, ^∗∗∗^ in case of *P*-value < 0.05, <0.01, and <0.001; respectively.

### Homology Based Modeling and Molecular Docking Study

Three-dimensional structures of nine USPs were created using the MODELLER tool of MPI bioinformatics toolkit^14^ (Zimmermann et al., 2018; Gabler et al., 2020) based on the templates identified using the HHpred tool. Crystal Structure of the Usp protein of *Mycobacteria* (PDB-5AHW_F), hypothetical protein MJ0577 (PDB-1MJH_B), UspA from *Lactobacillus plantarum* (PDB-3S3F_A), USP from *A. thaliana* (PDB-3GM3_E), USP from *Nitrosomonas* (PDB-3TNJ_A), USP from *Burkholderia pseudomallei* (PDB-4WNY_A), Human Cdkl5 Kinase Domain (PDB-4BGQ_A), Pseudokinase MLKL from *Mus musculus* (PDB-4BTF_A) and hypothetical protein PH0823 (PDB-2DUM_B) were used as templates for OsUSP2, OsUSP3, OsUSP6, OsUSP14, OsUSP22, OsUSP32, OsUSP33 and OsUSP36, respectively. All these structures were visualized using BIOVIA Discovery Studio v.21.1.0.20298 (BIOVIA, 2021) (Supplementary Figure 2).

**FIGURE 1:**
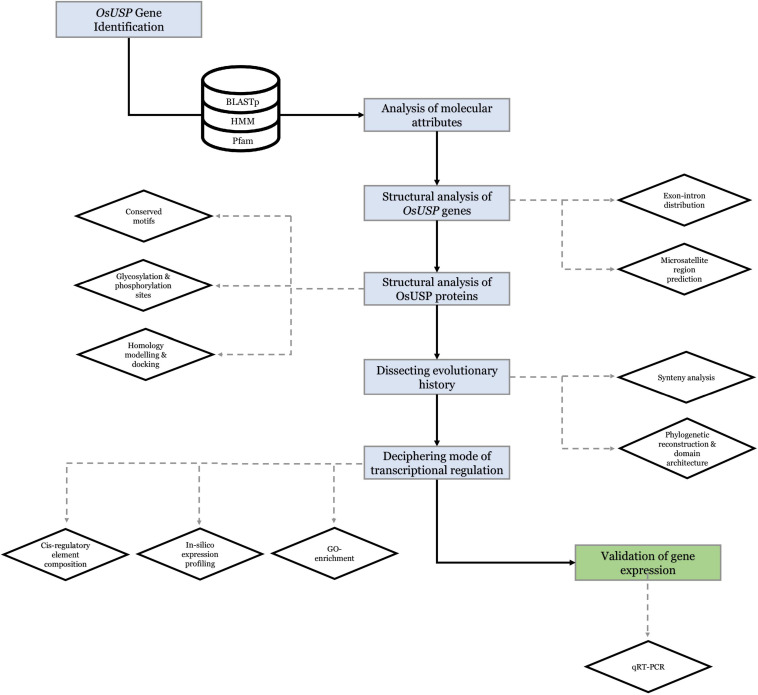
Strategic framework of the present study.

3D structure of the above nine OsUSP proteins was used as a receptor to find out the binding affinity with three well-established inhibitors of *E. coli* UspA *–* ZINC000104153710, ZINC000004268284, ZINC000000217308 (Bandyopadhyay et al., 2021). Four kinase inhibitors – fisetin, luteolin, myricetin, quercetin (Cassidy and Setzer, 2010) were used to check their binding affinity toward the kinase domain-containing OsUSPs. The 3-D chemical structure of these ligands was downloaded from the PubChem compound database^15^ (Kim et al., 2021) as an SDF file. The overall docking process was done using PyRx Virtual Screening software (Dallakyan and Olson, 2015) which uses Open Babel (O’Boyle et al., 2011) for importing SDF files, removing salts and energy minimization, uses AutoDock tools (Morris et al., 2009) for the preparation of protein and to generate input files and AutoDock Vina (Trott and Olson, 2010) as docking wizard. Grid box parameters were set to perform a blind docking (Hetényi and van der Spoel, 2002) of the inhibitors on the targeted proteins. The 2-D and 3-D interaction of the protein–ligands complex has been observed by using the BIOVIA Discovery Studio v.21.1.0.20298 (BIOVIA, 2021). Overall, a strategic framework of the present study has been summarized in Figure 1.

## Results

### Identification of *USP* Genes in Rice

Based on the blastp and HMM search, a total of 44 candidate *USP* genes were identified in rice (*Oryza sativa* L.). Relevant details about these genes are presented in Table 1. These identified *USP* genes were named from *OsUSP1* to *OsUSP44* in order of their chromosomal position. Among them, two splice variants were observed for genes *OsUSP1*, *OsUSP2*, *OsUSP11*, *OsUSP16*, *OsUSP20*, *OsUSP22*, *OsUSP25*, *OsUSP27*, *OsUSP30*, *OsUSP31*, and *OsUSP35*; whereas, *OsUSP12* had three splice variants. The length of deduced complementary DNA sequence (CDS) of *OsUSP*s ranged from 222 bp (*OsUSP31*) to 2817 bp (*OsUSP20*) long. Altogether, the forty-four *OsUSP* genes encoded for a total of 57 proteins. The protein length ranged from 74 aa (OsUSP31) to 939 aa (OsUSP20). Meanwhile, the protein weight varied greatly from the lowest of 17.22 kDa (OsUSP13) to the highest of 120.81 kDa (OsUSP41), and the predicted isoelectric point range from 4.74 (OsUSP30) to 10.39 (OsUSP37). *In silico* subcellular localization prediction indicated that *OsUSPs* were localized in the cytoplasm, mitochondria, nucleus, and chloroplast (Table 1).

**TABLE 1:** List of identified *OsUSP* genes along with their molecular attributes.

**Gene**	**Chr. no**	**Locus**	**Transcripts**	**CDS coordinate (5′ to 3′)**	**CDS (bp)**	**PP (aa)**	**Mass (kDa)**	**pI**	**Localization**
*OsUSP1*	Chr1	Os01g0170600	Os01t0170600-01	3645508–3648396	819	273	17.95	10.13	Mt^1,2^, Chl^1,2,3^
			Os01t0170600-02	3645512–3650691	483	161	5.01	10.13	
*OsUSP2*	Chr1	Os01g0303800	Os01t0303800-01	11245253–11246737	549	183	18.76	6.22	Cyt^1,2^, Chl^1^, Nu^1^
			Os01t0303800-02	1245276–11246714	549	183	18.76	6.22	
*OsUSP3*	Chr1	Os01g0511100	Os01t0511100-01	17990846–17993750	504	168	17.92	7.55	Chl^1^, Cyt^1,2^, Mt^1^
*OsUSP4*	Chr1	Os01g0581400	Os01t0581400-01	22538998–22542226	2298	766	84.03	7.98	Cyt^1,2^, Chl^1^, Nu^1^
*OsUSP5*	Chr1	Os01g0783500	Os01t0783500-01	33198608–33201733	789	263	28.79	4.99	Chl^1,3^, Mt^1^
*OsUSP6*	Chr1	Os01g0849600	Os01t0849600-01	36496934–36498132	489	163	18.03	6.52	Cyt^1,2^, Mt^1,2^, Chl^1,2^
*OsUSP7*	Chr1	Os01g0875300	Os01t0875300-00	37982651–37985429	945	315	25.28	9.99	Chl^1,2,3^, Cyt^2^, Nu^2^
*OsUSP8*	Chr2	Os02g0152300	Os02t0152300-01	2869670–2874062	2328	776	86.19	6.66	Nu^1,2^, Chl^1^, Cyt^1,2^, Mt^1,2^
*OsUSP9*	Chr2	Os02g0218400	Os02t0218400-01	6609459–6613930	2193	731	88.49	8.75	Nu^1,2^, Cyt^1,2^, Mt^2^
*OsUSP10*	Chr2	Os02g0218600	Os02t0218600-00	6621779–6623047	1092	364	95.56	8.47	Chl^1,3^, Mt^2^, Nu^2^, Cyt^2^
*OsUSP11*	Chr2	Os02g0705400	Os02t0705400-01	29113442–29114646	258	86	19	5.37	Cyt^1^, Mt^2^, Chl^2^
			Os02t0705400-02	29113445–29117170	555	185	19	5.37	
*OsUSP12*	Chr2	Os02g0707900	Os02t0707900-01	29260063–29265497	534	178	19.87	6.39	Chl^1,2^, Cyt^1,2^, Nu^1,2^, Mt^1,2^
			Os02t0707900-02	29260085–29265446	534	178	19.87	6.39	
			Os02t0707900-03	29258565–29260280	597	199	19.87	6.39	
*OsUSP13*	Chr2	Os02g0760500	Os02t0760500-01	32023322–32027150	489	163	17.22	6.75	Cyt^1,2^, Mt^2^, Chl^2^
*OsUSP14*	Chr2	Os02g0773200	Os02t0773200-01	32643551–32644836	498	166	17.88	8.82	Cyt^1^, Mt^2^, Chl^1^
*OsUSP15*	Chr2	Os02g0787200	Os02t0787200-01	33434089–33439102	2319	773	84.75	7.37	Nu^1,2^, Chl^1^, Cyt^1,2^
*OsUSP16*	Chr3	Os03g0241600	Os03t0241600-01	7483344–7487296	1959	653	70.89	7.41	Chl^1,2^, Nu^1^, Cyt^1,2^, Mt^1^
			Os03t0241600-02	7483349–7484709	762	254	43.47	9.92	
*OsUSP17*	Chr3	Os03g0305400	Os03t0305400-01	10839459–10840785	543	181	18.93	4.87	Chl^1,2^, Mt^2^, Cyt^1,2^
*OsUSP18*	Chr3	Os03g0344166	Os03t0344166-00	12820005–12820732	321	107	16.84	6.62	Cyt^1,2^, Mt^1,2^, Chl^2^
*OsUSP19*	Chr3	Os03g0750000	Os03t0750000-01	30901840–30903031	258	86	19.23	6.95	Cyt^1,2^, Chl^1,2^, Mt^2^
*OsUSP20*	Chr3	Os03g0839900	Os03t0839900-01	35312754–35317359	2817	939	101.27	4.99	Nu^1,2^, Mt^1^
			Os03t0839900-02	35312453–35317266	2817	939	101.27	4.99	
*OsUSP21*	Chr5	Os05g0157200	Os05t0157200-01	3345130–3347246	504	168	18	5	Cyt^1^, Chl^1^
*OsUSP22*	Chr5	Os05g0170200	Os05t0170200-01	4205610–4207693	390	130	21.65	10.08	Chl^1,2^, Cyt^1,2^, Mt^1,2^
			Os05t0170200-02	4205610–4207693	612	204	21.65	10.08	
*OsUSP23*	Chr5	Os05g0355400	Os05t0355400-01	16859727–16860956	333	111	18.71	7.83	Mt^2^, Chl^1,2^, Cyt^1,2^
*OsUSP24*	Chr5	Os05g0428400	Os05t0428400-00	21006679–21009677	678	226	24.08	10.01	Nu^1,2^, Chl^1^, Mt^2^
*OsUSP25*	Chr5	Os05g0453700	Os05t0453700-01	22276282–22278726	570	190	20.9	6.23	Cyt^1^, Chl^1^, Nu^1^, Mt^2^
			Os05t0453700-02	22276284–22278726	498	166	18.53	6.51	
*OsUSP26*	Chr5	Os05g0501700	Os05t0501700-01	24696226–24699271	807	269	29.82	5.74	Chl^1,2,3^
*OsUSP27*	Chr6	Os06g0140800	Os06t0140800-01	2140055–2146616	2421	807	90.05	6.58	Nu^1,2^, Cyt^1^, Chl^1^
			Os06t0140800-02	2140306–2142183	1281	427	90.05	6.58	
*OsUSP28*	Chr6	Os06g0191900	Os06t0191900-00	4641462–4644922	2436	812	86.41	8.01	Nu^1,2^, Chl^1^, Cyt^1,2^, Mt^2^
*OsUSP29*	Chr6	Os06g0574200	Os06t0574200-01	22271338–22275542	2421	807	88.28	8.11	Chl^1,2,3^, Nu^1,2^, Mt^1,2^
*OsUSP30*	Chr7	Os07g0551400	Os07t0551400-01	21894865–21898610	807	269	28.9	4.83	Chl^1,2,3^, Cyt^1^, Nu^1,2^, Mt^1^
			Os07t0551400-02	21897237–21898566	807	269	28.19	4.74	
*OsUSP31*	Chr7	Os07g0673400	Os07t0673400-01	28468527–28469554	516	172	17.96	7.01	Cyt^1,2^, Chl^1,2^, Nu^1^, Mt^2^
			Os07t0673400-02	28468619–28469554	222	74	17.96	7.01	
*OsUSP32*	Chr8	Os08g0249100	Os08t0249100-01	9101238–9105746	1806	602	67.33	6.51	Cyt^1,2^, Chl^1,2^, Nu^1,2^
*OsUSP33*	Chr9	Os09g0569800	Os09t0569800-00	22736559–22741162	2577	859	96.14	6.79	Chl^1,3^, Nu^2^, Cyt^2^
*OsUSP34*	Chr9	Os09g0570000	Os09t0570000-01	22749994–22752169	1083	361	81.92	5.98	Cyt^1,2^, Nu^1,2^
*OsUSP35*	Chr10	Os10g0100500	Os10t0100500-01	67119–72969	2436	812	90.78	7.15	Chl^1,3^, Nu^1,2^, Cyt^1,2^, Mt^2^
			Os10t0100500-02	71174–72765	972	324	90.78	7.15	
*OsUSP36*	Chr10	Os10g0437500	Os10t0437500-01	15663637–15664570	546	182	18.88	5.87	Cyt^1,2^, Chl^1,2^
*OsUSP37*	Chr10	Os10g0463300	Os10t0463300-01	17074150–17075296	621	207	21.48	10.39	Chl^1,2^, Cyt^1^, Nu^1,2^
*OsUSP38*	Chr10	Os10g0561500	Os10t0561500-01	22136957–22141731	2349	783	86.88	7.06	Cyt^1,2^, Chl^1,3^, Nu^1,2^
*OsUSP39*	Chr11	Os11g0194900	Os11t0194900-01	4734980–4738431	2004	668	80.54	7.66	Chl^1,2,3^, Cyt^1,2^
*OsUSP40*	Chr12	Os12g0180500	Os12t0180500-00	4056054–4058982	2190	730	78.57	7.48	Chl^1,2^, Mt^1,2^, Cyt^1^
*OsUSP41*	Chr12	Os12g0181200	Os12t0181200-00	4091707–4097797	1176	392	120.81	7.97	Chl^1^, Mt^1,2^, Cyt^1^, Nu^2^
*OsUSP42*	Chr12	Os12g0501400	Os12t0501400-01	19089135–19092961	678	226	24.24	10.28	Chl^1,2^, Nu^2^
*OsUSP43*	Chr12	Os12g0552400	Os12t0552400-01	22427863–22429178	483	161	17.54	7.36	Cyt^1,2^, Chl^1^, Nu^2^, Mt^2^
*OsUSP44*	Chr12	Os12g0552500	Os12t0552500-01	22429432–22431789	510	170	18.42	7.28	Cyt^1,2^, Chl^1,2^, Nu^1,2^, Mt^2^

*^1^WoLFPSORT, ^2^CELLO subcellular localization prediction tool, ^3^ChloroP. Mt, mitochondria; chl, chloroplast; Nu, nucleus; Cyt, cytoplasm.*

### Analysis of *OsUSP* Gene Structure, Conserved Motifs and Amino Acid Content

**FIGURE 2:**
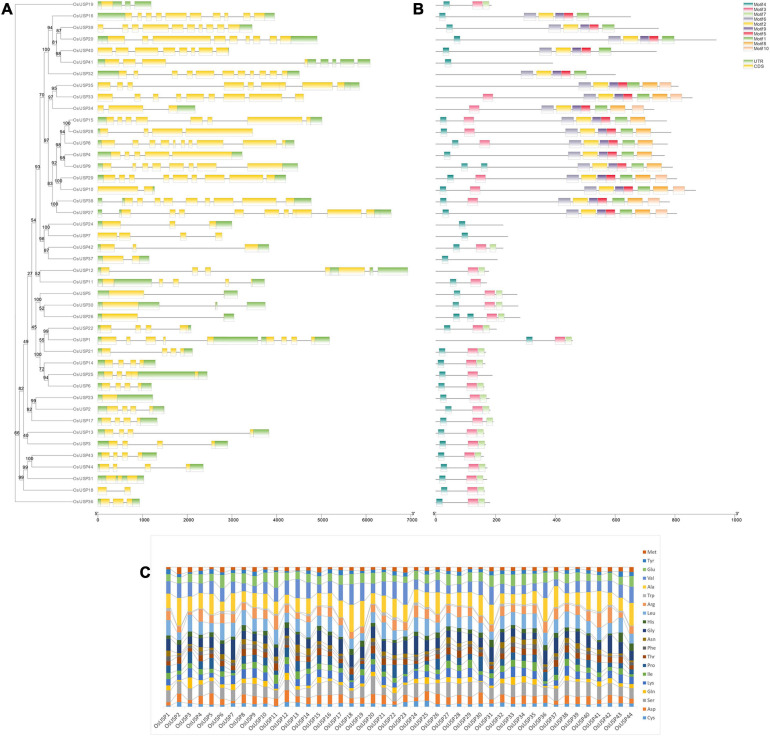
Overview of gene structure, conserved motifs, and amino acid composition of OsUSP members. **(A)** Exon–intron distribution of all the identified *OsUSP* genes. Yellow and green boxed indicate exon and UTR regions, respectively, whereas black lines indicate the position of introns. **(B)** The position of conserved motifs in OsUSPs. Each colored box indicates a single motif (legends are provided in the figure). The two scales at the bottom indicate gene (left) and protein (right) length. A maximum-likelihood tree with 1000 ultrafast bootstrap replicates was constructed using IQ-Tree. The bootstrap values are given as numbers on the internal nodes. **(C)** Amino acid (aa) composition of OsUSPs shown in a stacked bar plot. The percent content of each amino acid is indicated with a different color (see figure for details).

To better understand the development of the *OsUSP* gene family, the exon–intron distribution of *OsUSP* genes was analyzed. In general, the number of exons and introns in *OsUSP* genes showed moderate variation (Figure 2A). In the case of introns, the numbers ranged from zero to ten, with *OsUSP32* having the highest and *OsUSP23* having the lowest number of introns (10 and 0, respectively). On average, *OsUSP* genes that contained both USP and kinase domains had a higher number of introns compared to those with only a USP domain. This indicates a similarity in the exon–intron architecture between genes that were more similar on a protein level. Similarly, the number of exons varied from one to eleven, with *OsUSP32* having the highest number of exons. Only a single exon was found in four genes, namely – *OsUSP19, OsUSP23, OsUSP26*, and *OsUSP30.* Several *OsUSP* genes such as *OsUSP1, OsUSP12, OsUSP25, OsUSP31* contain multiple 3′-UTR regions. This likely reflects the possibility of alternative splicing in these genes.

Elucidation of conserved motifs using MEME software revealed a total of 10 conserved motifs among rice USP proteins (Figure 2B). As expected, there was quite some variation in terms of motif number in each protein. In general, most proteins either contained three, nine or all 10 of the conserved motifs. This observation perfectly coincided with the presence of all three domains – USP, Kinase, and U-Box; two domains – USP and Kinase; or just a single USP domain in the protein. In the former case, 10 motifs were found in the proteins, whereas for those with only the USP domain, the number of conserved motifs was between one to three. From this observation, it could be deduced that motifs 3, 4, and 7 are associated with the USP domain; motifs 1, 2, 5, 6, 8, and 9 are linked to the kinase domain and motif 10 corresponds to the U-box domain. In addition to this, the amino acid (aa) composition of OsUSPs (Figure 2C) showed that OsUSPs with the same domain(s) show more similarity in their aa pattern compared to those with slightly different domain attributes. This can be exemplified by looking at the aa composition of USP 32-39 (Figure 2C). Here, USP 32-35 and 38-39 have both a USP and a kinase domain and are relatively alike in their aa composition. However, USP36 and USP35 lack a kinase domain and it is discernible that their aa composition is more diverse from USP 32-35 and 38-39. There appears to be a consistency in gene structure, motif conservation, and AA composition with the type of domain a given OsUSP contains.

### Chromosomal Distribution and Synteny Analysis

**FIGURE 3:**
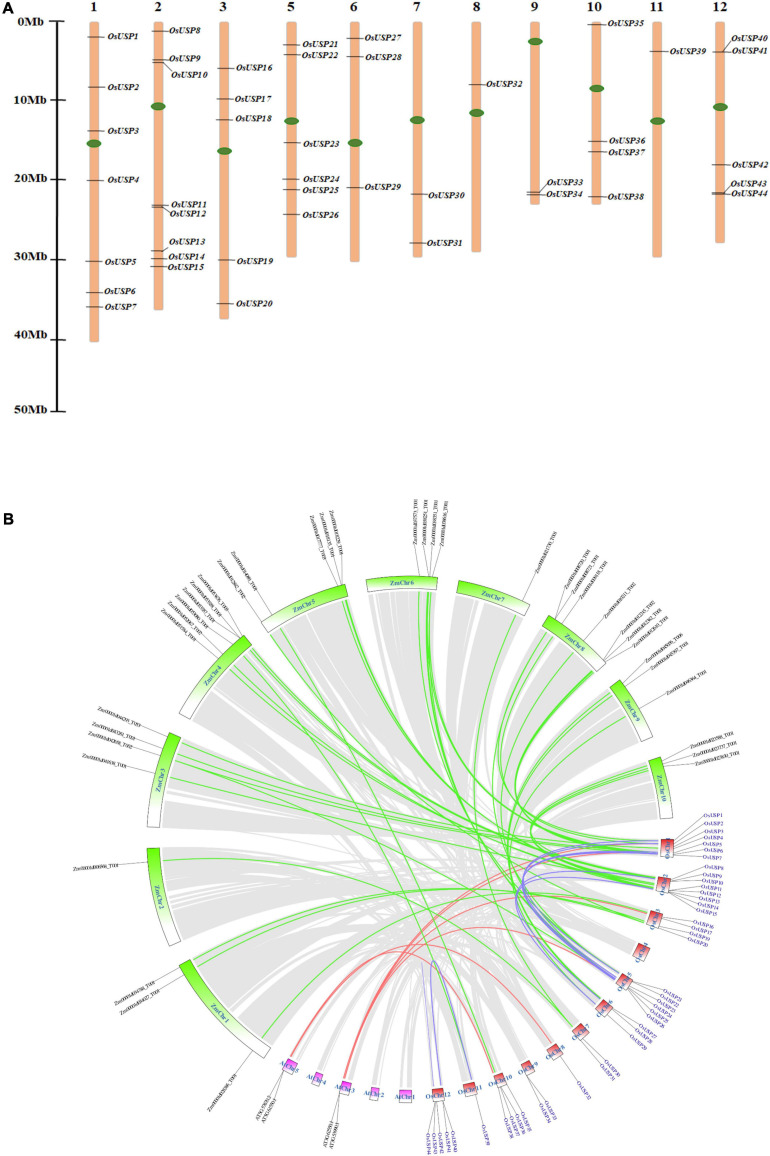
Chromosomal localization and syntenic relationship of rice *USP* genes. **(A)** Distribution of 44 *OsUSP* genes over 11 rice chromosomes. Each *OsUSP* gene was pointed to the exact position of a particular chromosome that could be calculated by using the scale provided on the left. Each chromosome number is indicated on the top of each chromosome bar. Maximum eight genes clustered in chromosome 2, while minimum one gene was present on chromosome no 8 and 11. The left bar represents scale in Megabase (Mb). **(B)** Synteny analysis of the rice genome with itself, with *Arabidopsis thaliana* and with maize genomes. Synteny blocks corresponding to *OsUSP* genes were indicated by different colors. Blue lines indicate synteny between *OsUSP* genes, the red line indicates synteny between *OsUSP* genes and their orthologs in *Arabidopsis thaliana*, and green lines indicate synteny between *OsUSP* genes and their orthologs in maize. Chromosomes of the three species were shown in different colors – red, pink, and green represent rice, *Arabidopsis thaliana*, and maize chromosomes, respectively. Positions of all *OsUSP* genes (blue) and their counterparts in two other species (black) were indicated as well.

The identified *OsUSP* genes were distributed over 11 out of 12 rice chromosomes (Figure 3A). The exact number of *OsUSP* genes per chromosome varied widely. Chromosome 2 contained the highest number of eight *USP* genes, followed by chromosome 1 with seven genes. A relatively moderate number of *USP* genes were found in chromosomes 5 and 12, where the number reached six and five, respectively. Both chromosomes 3 and 10 contained four members. As for other chromosomes, the number of *USP* gene family members ranged from one to three, with chromosome 4 being the only exception, which did not contain any *USP* genes.

The gene duplication events among *OsUSP* genes were investigated to evaluate the extension of this gene family members. A total of 11 *OsUSP* gene pairs were found to be duplicated- *OsUSP1* and *OsUSP22*, *OsUSP2* and *OsUSP23*, *OsUSP2* and *OsUSP17*, *OsUSP4* and *OsUSP29, OsUSP5* and *OsUSP26, OsUSP6* and *OsUSP25, OsUSP7* and *OsUSP24*, *OsUSP9* and *OsUSP29, OsUSP15* and *OsUSP28, OsUSP18* and *OsUSP31*, and *OsUSP2* and *OsUSP23* (Supplementary Table 6). All of them are possessing segmental type of duplication, except one with tandem type. A negative selection was observed for all the duplicated pairs except one with the ratio of nonsynonymous (Ka) to synonymous (Ks) values more than one. The calculated divergence time of the duplication was varied from 28 to 150 Mya (Supplementary Table 6).

Chromosomal synteny analysis was carried out to understand the expansion and diversification of *USP* genes within the rice genome and to compare with other monocot (maize) and dicot (*Arabidopsis*) organisms (Figure 3B). Both segmental and tandem duplication were visible for *USP* genes within the rice genome, with the former dominating the latter. A total of sixteen paralogous *USP* genes were recognized within the rice genome that were localized on chromosomes 1, 2, 5, 6, 11, and 12. Whereas, a single tandem duplication was detected on chromosome 12 between *OsUSP43* and *OsUSP44*. Interesting observations came to light during the syntenic comparison of *OsUSP* genes with *Arabidopsis* and maize. Starting with the dicot genome, only four *OsUSP* orthologs were detected in the *Arabidopsis* genome and were limited to chromosomes AtChr3 and AtChr5 (Figure 3B). In contrast to this, a relatively large number of *OsUSP* orthologs were detected in the monocot plant, maize. In total, 37 orthologous genes were spread throughout all maize chromosomes (Figure 3B). This likely indicates that *OsUSP* genes have diverged more from their counterparts in the *Arabidopsis* genome compared to the more closely related monocot, maize. Overall, this indicates that both duplication and intense diversification have played a characteristic role in the evolution of the *OsUSP* gene family.

### Phylogeny of USPs From Three Species and Their Domain Architecture

**FIGURE 4:**
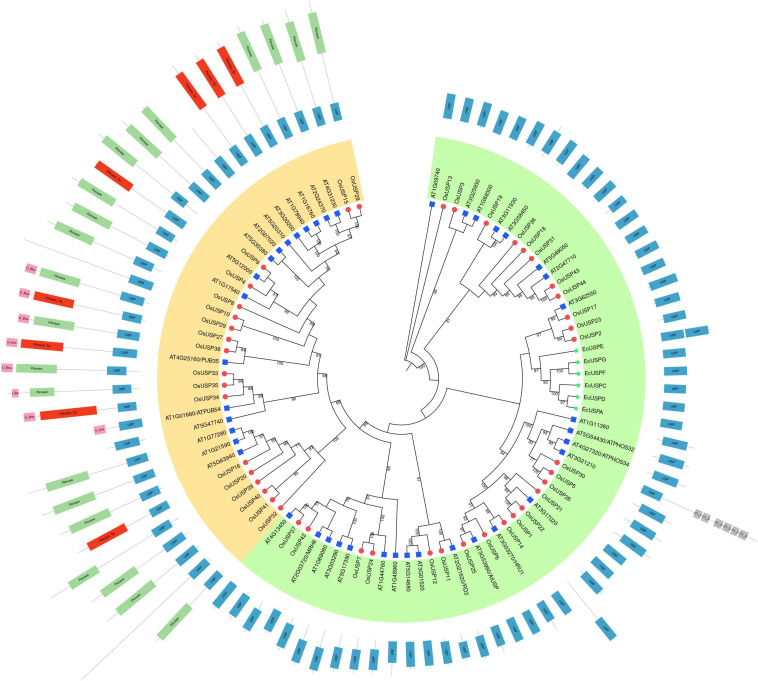
Phylogenetic relationship between rice, *Arabidopsis thaliana*, and *E. coli* USPs. A maximum-likelihood tree with 1000 ultrafast bootstrap replicates was produced using IQ-Tree. The final tree was visualized and edited in iTOL. USP members from the three different species are specified using different colored shapes – blue rectangles, red circles, and green stars sequentially specify *Arabidopsis thaliana*, *Oryza sativa*, and *E. coli* USPs. Overall, USPs were sub-grouped into two clusters – green and yellow based on the domain architecture of each protein. The green clusters exclusively contain protein that harbors only a USP domain. As for the yellow cluster, almost all members harbor an additional kinase or U-Box domain or both (except a few). Information regarding the presence and position of a domain was added in iTOL and the name of each domain is indicated within the corresponding boxes.

To explore the evolutionary pattern of USP proteins in three species – rice, *Arabidopsis*, and *E. coli*, we carried out a phylogenetic analysis using their all USP members (Figure 4). The addition of domain architecture of these proteins to the tree showed two clear clusters – one with members that contain only a USP domain (Figure 4, green cluster) and the other with members that mostly contain an additional kinase domain on top of the USP domain (Figure 4, yellow cluster). Overall, the number of proteins with only a USP domain was higher than the other type with more structural diversity in terms of their domain architecture. Focusing on the yellow cluster, most members had a kinase domain in addition to the characteristic USP domain. For some members, the presence of a U-box domain can also be seen. Interestingly, two rice USPs that lack a kinase domain (OsUSP10 and 41) were also found to group in this cluster. With respect to comparison of USP evolution between species, all *E. coli* USPs grouped together in the first cluster (green). A closer inspection of the tree showed that USPs usually clustered closest to a paralog from the same species rather than its ortholog from another species. This is because only in a relatively small number of cases can clear orthologs between the two species be identified in the tree in Figure 4. For instance, clear orthologs in *Arabidopsis* could only be discerned for rice USPs – 9, 11, 14, 17, 25, and 37.

### *In silico* Identification of SSRs, and Prediction of Glycosylation and Phosphorylation Sites

Molecular markers allow the identification of genes of interest within a particular location. A major concern of genome analysis is to explore molecular markers related to genetic factors underlying observable traits. Forty-four *OsUSP* genes with all splice variants were analyzed for SSR markers. Out of 61 sequences, 19 SSR markers were distributed among 15 sequences. The most abundant were trinucleotide repeats accounting for 89.47% (17 occurrences), followed by mononucleotide and dinucleotide repeats both with one occurrence accounting for 5.26% of all SSRs, no tetra, penta or hexanucleotide repeats were found. The dominant SSR motif was GCG with a frequency of 47.36 with nine occurrences followed by other mono, di and trinucleotide repeats (Supplementary Table 7). Higher repeat numbers were observed in trinucleotide SSRs. Several sequences possessing more than one SSR marker were found three. Furthermore, glycosylation plays a key role in secondary protein processing for the functioning of protein within cells and in determining protein structure, function, and stability. In this study, 25 out of 44 OsUSPs have shown one to many predicted glycosylation sites with the threshold of 0.5. A total of 68 glycosylation sites have been predicted in 25 OsUSP proteins. The highest number of glycosylation sites was found in OsUSP8 with nine sites followed by OsUSP10, OsUSP32, OsUSP35 (five sites) and OsUSP27, OsUSP33, OsUSP38 with four sites. *N*-Glycosylation score greater than 0.5 and jury agreement 9/9 or potential >0.75 indicates high specificity of glycosylation site. Among the total of 68 predicted sites, 19 sites in 13 OsUSP proteins have the potential score greater than 0.5 and jury agreement 9/9, and thus, have the highest chance of glycosylation mediated stable structure formation (Supplementary Table 8). Protein phosphorylation is the another most significant type of post-translational modification of cells in which protein kinases phosphorylate an amino acid residue, mostly Tyr, Thr, Ser in the case of eukaryotes by adding covalently bound phosphate groups. A significant number of phosphorylation sites were predicted in all the members of OsUSP (Supplementary Table 9). The highest number of phosphorylation sites were predicted in OsUSP20, followed by OsUSP29, OsUSP27 and OsUSP39. Predicted results also showed that serine (S) sites were more phosphorylated than threonine (T) and tyrosine (Y) sites. As for the types of kinases, the prevalence of the predicted ones was not specific, while PKC was the most common.

### Analysis of Expression Pattern of *OsUSP* Genes During Developmental Stages and in Various Tissues of Rice

**FIGURE 5:**
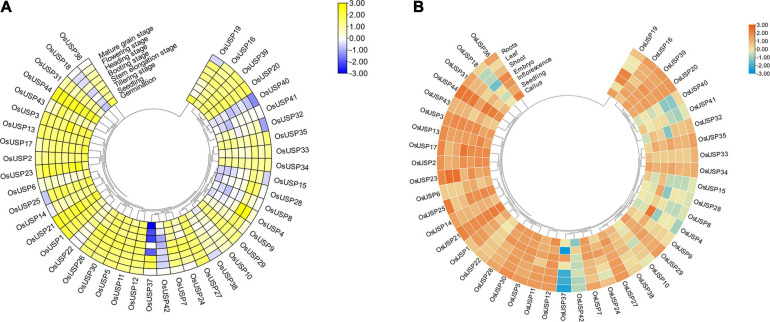
Overview of the developmental stage and tissue-specific expression profile of *OsUSP* genes. **(A)** Expression pattern of each *OsUSP* gene during multiple developmental stages of rice plants. **(B)** Expression pattern of *OsUSP* genes in different tissues of rice plants. The developmental stage and tissue-specific expression data were downloaded from Genevestigator using the corresponding gene IDs of rice *USPs*. Raw expression levels were log10-tranformed and visualized with “Heatmap Illustrator” option in TBtools (v.1.092). The scale indicates the magnitude of gene expression. The phylogenetic tree is same as the one shown in Figure 2.

To get a notion about the magnitude of *OsUSP* genes expression, we carried out an expression analysis using previously generated transcriptomics data for *OsUSP* genes during different developmental stages (Figure 5A) and anatomical tissues (Figure 5B). Most of the *OsUSP* genes consistently show high levels of expression during all the developmental stages (Figure 5A) except *OsUSP18, OsUSP40, OsUSP41*, and *OsUSP42.* These genes show relatively low expression levels in almost all stages. However, some interesting candidates show preferentially high expression during certain stages. For instance, *OsUSP15* is highly expressed during the flowering and heading stage but less prominent during germination, seedling, tillering, and mature grain stage. A similar observation is true for *OsUSP8* and *OsUSP37* with the latter showing a stronger contrast in expression.

Moving onto expression profiles in tissues, a similar pattern was observed where most *OsUSP* genes exhibited moderate to high expression levels in all tissues (Figure 5B), with exception of *OsUSP4, OsUSP8, OsUSP15, OsUSP18, OsUSP28, OsUSP37, OsUSP40, OsUSP41*, and *OsUSP42.* Some genes showed a mixed pattern, while some tissues are showing higher expression for few genes than others. *OsUSP4* showed very high expression in inflorescence tissues but moderate to low level of expression in others. Comparably, *OsUSP37* had a very low expression in roots, shoots, leaves, and seedlings but a high expression in inflorescence tissues. Overall, most of *OsUSP* genes showed an appreciable level of expression both during different developmental stages and tissues of the plant.

### Analysis of Stress-Specific Expression Pattern of *OsUSP* Genes and Their *Cis*-Regulatory Elements

**FIGURE 6:**
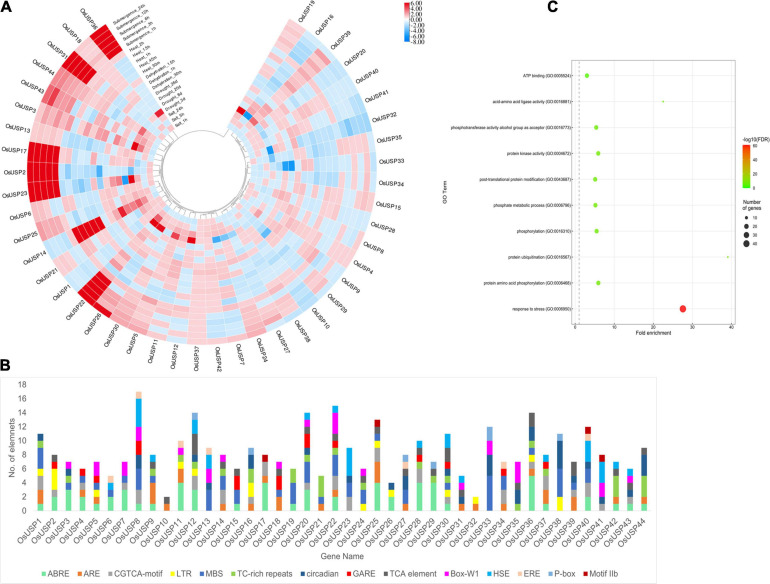
Overview of the stress-specific expression profile of *OsUSP* genes, the composition of their promoter regions, and functional annotation. **(A)** Modulation of *OsUSP* gene expression under several abiotic stress conditions – submergence, heat, dehydration, drought, and salt stress. Expression data of rice *USP* genes under multiple abiotic stresses were downloaded from Genevestigator using the corresponding gene IDs. Change in the expression pattern was calculated in terms of log_2_-fold change. The scale on the right indicates the magnitude of up or downregulation. For some genes, the amount of fold change was higher (or lower) than indicated by the scale. However, to maintain uniformity in the colors of the boxes the scale was limited to +6 and –8 manually. The phylogenetic tree is similar to the one shown in Figure 2. **(B)** The presence of different *cis*-regulatory elements in the 1000 bp – upstream region of *OsUSP* genes is shown using a stacked bar plot. Each color indicates a different *cis*-regulatory element (see figure for legends) and the length of the bar designates the number of elements. Several stress-responsive *cis*-regulatory elements were visible including – ABRE, ARE, MBS, LTR, HSE, P-box, etc. **(C)** GO-enrichment analysis on *OsUSP* genes showed enrichment for stress response in over 40 USP genes. The size of the circles indicates the number of gene and the color corresponds to the –Log_10_(FDR) values.

To evaluate the role of *OsUSP*s in stress conditions, analysis of the expression data of all the identified *OsUSP* genes were performed under multiple stress conditions including – heat, salt, drought, dehydration, and submergence (Figure 6A). Expectedly, most *OsUSP* genes showed strong upregulation or downregulation in response to these adversities. However, the number of upregulated genes was slightly higher than those that were downregulated. Some *USP* genes including – *OsUSP2, OsUSP17, OsUSP22, OsUSP23, OsUSP26, OsUSP31*, and *OsUSP36* were strongly upregulated (even higher than 6-fold) under submergence conditions. All genes (except *OsUSP26*) showed a strong upregulation from the early onset of submergence. Some genes such as *OsUSP1, OsUSP10, OsUSP16, OsUSP20, OsUSP28, OsUSP42* showed downregulation as well. However, the magnitude of downregulation was not as strong for those that were upregulated. In the case of heat stress, the expression of *OsUSP* genes varied quite a lot. The strongest upregulation was detected for *OsUSP14*, whereas some genes showed both upregulation and downregulation in a temporally separated fashion like *OsUSP15, OsUSP22, OsUSP30*, etc. Moving on to dehydration and drought stress, upregulation of several USP genes was more prominent at the early onset of stress application. For instance, genes *OsUSP3, OsUSP5, OsUSP11, OsUSP14, OsUSP19, OsUSP22, OsUSP23, OsUSP26*, and *OsUSP36* showed considerable upregulation at the earlier stages of drought stress, but their expression varied in the later time points. Some of these genes such as *OsUSP5* and *OsUSP14*, showed upregulation in both conditions and at all time points. There was also a mixed response of *OsUSP* genes in response to salt, with some being upregulated and others downregulated. The magnitude of upregulation was stronger mostly at the longer period of salt application (24 h). In terms of consistency across all stress conditions, the expression profile of *OsUSP2, OsUSP3, OsUSP22, OsUSP32, OsUSP33, OsUSP39*, and *OsUSP44* was very interesting. In general, *OsUSP3, OsUSP22, OsUSP39*, and *OsUSP44* were mostly upregulated and *OsUSP2, OsUSP32*, and *OsUSP33* were mostly downregulated across the conditions (Figure 6A).

Additionally, we checked the composition of the putative promoter region of *OsUSP* genes concerning the presence of different *cis*-regulatory elements. After surveying the 1000 bp-upstream sequence of each gene, most rice *USP* promoters harbored numerous *cis*-regulatory elements which are known to be involved in stress-induced gene regulation (Figure 6B). The motifs that were most commonly found in the *OsUSP* promoter regions were – ABRE, ARE, LTR, MBS, and TC-rich repeats, which are recognized for attracting stress-specific transcription factors (TFs). To add to this line of evidence, a GO enrichment analysis on all the *OsUSP* genes was performed (Figure 6C). This showed that out of the 44 genes identified, 41 *OsUSP* genes were annotated to be involved in stress response (GO:0006950). In short, the stress-specific modulations of rice *USP* genes are clear.

### Validation of Gene Expression Profile of Nine Selected *OsUSP* Genes in Response to Abiotic Stress

**FIGURE 7:**
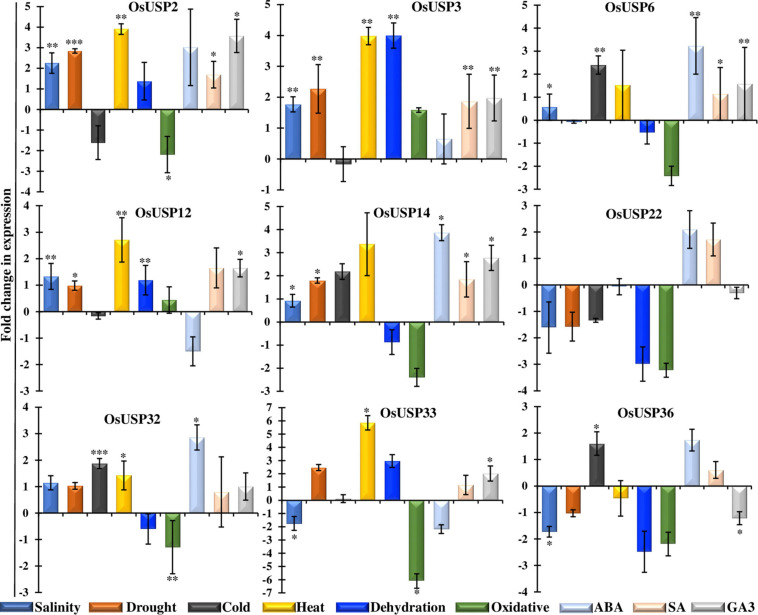
Functional validation of selective *OsUSP* genes expression in response to various abiotic stresses and hormonal treatments by qRT-PCR. Expression profiles of nine *OsUSP* genes were performed in response to salinity, drought, cold, heat, dehydration, and oxidative stresses, and ABA, SA and GA3 treatments in BRRI-53 rice variety. A scale showing Log_2_-fold change of each gene under each condition was presented at the left. The results were represented by the mean value of fold change ± standard deviation using rice *eEF1* gene as a reference. Statistical significance was determined using two-tailed paired Student’s *t*-test and are represented with ^∗^, ^∗∗^, ^∗∗∗^ for a *P*-value < 0.05, <0.01, and <0.001; respectively.

As an expression profile of genes under stress condition is important to understand their function, it is crucial to assess mRNA-seq expression patterns experimentally to ensure consistency. Thus, we carried out quantitative RT-PCR analysis of nine selected *OsUSP* genes in leaf tissue after 16 h of abiotic stress treatments including salt, drought, cold, heat, dehydration, H_2_O_2_ and hormonal treatment – ABA, SA, and GA3. The real-time PCR expression profile of the selected genes (Figure 7) reveal a good correlation with the mRNA-seq data (Figure 6A). Among nine genes, *OsUSP2, OsUSP3, OsUSP6, OsUSP12*, *OsUSP14, OsUSP32, OsUSP33* showed considerable upregulation in heat stress whereas *OsUSP22* and *OsUSP36* showed downregulation. Consistent with the mRNA-seq expression data, *OsUSP14* and *OsUSP22* genes showed a similar pattern of expression under heat stress. Under salinity stress *OsUSP2*, *OsUSP3*, *OsUSP6, OsUSP12*, *OsUSP14* and *OsUSP32* showed low to medium upregulation while *OsUSP22*, *OsUSP33* and *OsUSP36* showed downregulation in rice leaves. Some genes such as, *OsUSP2*, *OsUSP22* and *OsUSP32* showed differential expression patterns in salt stress which is different from the mRNA-seq data. However, the expression level of *OsUSP3* and *OsUSP33* were consistent with the expression pattern mRNA-seq data. Furthermore, the qRT-PCR results revealed that the expression level of *OsUSP2*, *OsUSP3*, *OsUSP12*, *OsUSP14*, *OsUSP32*, *OsUSP33* was upregulated under drought stress. Apart from that, *OsUSP6* showed no fold change, compared to control under drought stress. In dehydrated condition, only *OsUSP2*, *OsUSP12* and *OsUSP33* showed reasonable upregulation, while *OsUSP14* showed downregulation. The occasional inconsistency of gene expression of the selected genes between mRNA-seq and RT-PCR might be due to the differences in sampling time following stress/hormone treatment or the genotypic difference between the rice japonica variety and the BRRI-53 variety.

Expression analysis of nine selected *OsUSP* genes were further conducted in other abiotic treatments such as cold, oxidative, and hormonal stress. In cold treatment, *OsUSP* genes showed low to medium upregulation in leaf samples. *OsSUP6* does not show any expression in drought stress but is highly expressed in cold stress whereas *OsUSP33* showed negligible up-regulation which is close to control. *OsUSP14*, *OsUSP32*, *OsUSP36* showed up-regulation under cold stress. But we observed that the other *USP* genes such as *OsUSP2*, *OsUSP3*, *OsUSP12*, *OsUSP22* and *OsUSP36* were downregulated in cold. *OsUSP*s showed down-regulation in oxidative stress except *OsUSP3* and *OsUSP12*, which need to be investigated further. Furthermore, *OsUSP* showed a differential regulation in rice leaf tissue under hormonal influences like ABA, SA and GA3 (Figure 7). The qRT-PCR results suggested that most of the *USP* genes such as *OsUSP2*, *OsUSP3*, *OsUSP6*, *OSUSP14* and *OsUSP32* up-regulated in all the hormonal stresses. Apart from that, *OsUSP12* and *OsUSP33* showed upregulation but downregulated in ABA stress. The varying responses of *OsUSPs* could thus be attributed to different abiotic and hormonal stresses.

### Molecular Docking Analysis of Selected OsUSPs

**FIGURE 8:**
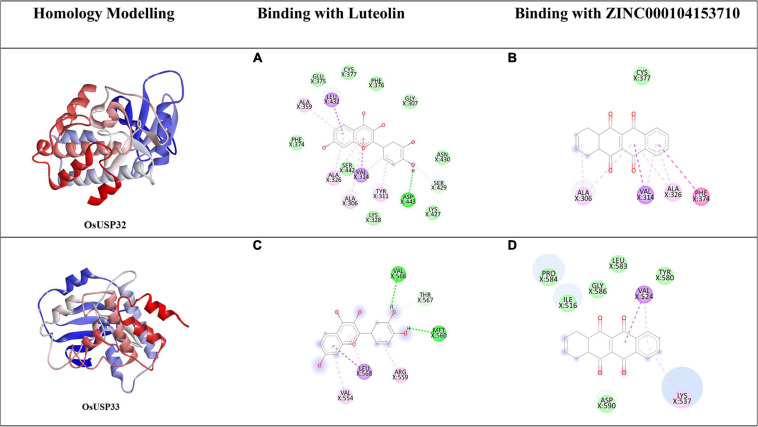
Homology modeling and molecular docking of two OsUSP (OsUSP32, OsUSP33) proteins having both USP and Pkinase domain. The first column represents the predicted 3-D structure, the second and third column represents the 2-D interaction of each protein with Luteolin **(A,C)** and ZINC000104153710 **(B,D)**, respectively. The green, light green, pink, violet, purple represent residues involved in the hydrophobic interactions, carbon-hydrogen bond interactions, Pi-Pi-T shaped, Pi-Sigma and Pi-Alkyl interactions, respectively.

Among 44 OsUSP proteins, 9 OsUSPs showed significant up and down-regulation to various stresses and were selected as receptors for docking study to check their affinity toward various inhibitors (Table 2). Of these 9, only two (OsUSP32, OsUSP33) have both USP and kinase domain, docked against UspA and PKC inhibitors. Rest seven contain only the USP domain, therefore, they were docked against only UspA inhibitors. All these selected nine proteins showed significant affinity toward UspA inhibitors (ZINC000104153710, ZINC000004268284, ZINC000000217308), and both OsUSP32 and OsUSP33 showed affinity toward four kinase inhibitors (Fisetin, Luteolin, Myricetin, Quercetin) (Table 2). Among the three USP inhibitors the highest affinity was found for Zinc000104153710 and in the case of four kinase inhibitors, Luteolin showed the highest affinity. Among all nine selected proteins, OsUSP32 showed the highest affinity toward UspA inhibitor Zinc000104153710 and PKC inhibitor luteolin with a binding energy of −9.6 kcal/mol and −9.2 kcal/mol, respectively, followed by OsUSP33 (−8.5 kcal/mol, −8.2 kcal/mol, respectively). The protein-ligand interaction of docked complexes was visualized using the Discovery Studio program (Figures 8A–D). The best-scored protein OsUSP32 with the highest binding affinity toward Zinc000104153710 formed one hydrogen bond with Asp443 having a distance of 2.21 Å, two pi-sigma bond with Val324 (3.93 Å), Leu432 (3.88 Å), one pi-alkyl bond with Ala359 (5.44 Å) and five alkyl bond with Ala306 (5.49 Å), Tyr311 (4.01 Å), Val314 (5.50 Å), Ala326 (4.62 Å, 5.06 Å) to stabilize within the binding pocket (Figure 8B). The highest binding affinity toward Luteolin was showed by OsUSP32 by forming one pi-pi-T-shaped bond with Phe374 (4.32 Å), two alkyl bond, one pi-alkyl bond, one pi-sigma with Ala306 (4.53 Å, 4.24 Å), Ala326 (5.35 Å), Val314 (4.43 Å), respectively (Figure 8A).

**TABLE 2:** Predicted binding affinity (kcal/mol) of selected OsUSP proteins against known USP and kinase inhibitors.

**OsUSP**	**Binding affinity toward USP**			**Binding affinity toward kinase**				**Center grid box (points in *X*, *Y*, *Z*-axis)**	**Dimensions (points in *X*, *Y*, *Z*-axis)**
	**inhibitors (kcal/mol)**			**inhibitors (kcal/mol)**					
	**ZINC00010** **4153710**	**ZINC00000** **4268284**	**ZINC000000** **217308**	**Fisetin**	**Luteolin**	**Myricetin**	**Quercetin**		
OsUSP2	−7.6	−0.6.6	−7.3	Not performed				12.9957 × 40.7169 × 20.2698	42.9413 × 54.5348 × 38.3895
OsUSP3	−6.9	−6.5	−7.1	Not performed				12.2149 × 14.507 × 30.1618	51.9887 × 39.8425 × 42.0554
OsUSP6	−9.0	−7.1	−8.8	Not performed				7.8863 × 3.4420 × 36.2376	57.7571 × 53.4284 × 34.9141
OsUSP12	−8.7	−7.2	−8.1	Not performed				25.4699 × 7.9124 × 21.9120	56.2974 × 44.9545 × 47.3260
OsUSP14	−8.4	−8.4	−8.6	Not performed				19.775 × 16.9896 × 5.4766	50.7828 × 52.9327 × 42.3257
OsUSP22	−7.8	−7.5	−7.5	Not performed				56.0183 × 11.0732 × 11.3009	42.7089 × 59.3073 × 69.2370
OsUSP32	−9.6	−8.5	−8.9	−8.3	−9.2	−8.1	−8.1	44.0062 × 32.1862 × 65.9105	60.2972 × 44.9794 × 57.9650
OsUSP33	−8.5	−7.4	−8.2	−7.7	−8.2	−7.6	−7.7	15.8940 × 15.2491 × 33.7449	48.3649 × 60.8299 × 58.4618
OsUSP36	−8.3	−8.6	−7.7	Not performed				22.4725 × 73.5770 × 1.2069	46.4691 × 47.2079 × 54.9569

## Discussion

Universal stress proteins have been shown to be induced under various abiotic stresses. To date, a diverse number of *USP* gene family have been identified from different plant species including, *Arabidopsis* (Bhuria et al., 2019), *Brassica napus*, *Triticum aestivum*, *Brassica rapa*, *Solanum lycopersicum*, *Solanum tuberosum*, *Oryza sativa japonica*, *Vitis vinifera*, *Zea mays* (Chi et al., 2019), *Malus sieversii* (Ledeb.) (Yang et al., 2019), Barley (Li et al., 2010), Pigeon pea (Sinha et al., 2016) etc. Usually, plant genomes harbor around twenty to fifty genes (Gou et al., 2020). However, more than a hundred members have been reported in the genome of *Brassica napus* and *Triticum aestivum* (Li et al., 2010; Chi et al., 2019). In the present study, a total of 44 *OsUSP* genes (Table 1) were identified in the rice genome.

The identified *USP* genes are spread throughout 11 out of 12 rice chromosomes (Figure 3A). The number of genes is slightly higher than previously reported (Bhowmick, 2019; Chi et al., 2019). Investigation into the structural properties of the identified genes and their corresponding protein showed that domain architecture played an important part in this. Analysis of synteny between rice *USP* genes showed that 16 out of 44 genes had traces of segmental duplication whereas two genes on chromosome 12 were likely derived from a tandem duplication event (Figure 3B). Thus, it appears that both segmental and tandem duplication played a part in the expansion of the *USP* gene family in rice. Further investigation into the syntenic relationship between rice *USP* genes and the genome of *Arabidopsis thaliana* and *Zea mays* (maize) revealed the presence of a higher number of orthologs in the monocot maize genome compared to the dicot *Arabidopsis thaliana* (Figure 3B). This indicates greater diversification of *USP* genes between the two angiosperms lineages – monocots and dicots compared to divergence within each lineage. In this regard, our study suggests that both duplication and diversification have played a major role in the evolution of the *USP* gene family in plants.

Analyses of the OsUSPs phylogeny revealed two clusters that finely coincides with the domain architecture of the proteins. The cluster (green) that groups with *E. coli* USPs likely represents those members that originated earlier in the evolutionary history of the *USP* gene family. In contrast, members from the other cluster (yellow) showed longer peptide lengths and the presence of a functional kinase domain. However, there were a few exceptions here, like *OsUSP10* and *OsUSP41*. Two *USP* genes could have undergone mutations that have rendered an existing kinase domain non-functional, or they could be on the verge of developing a kinase domain. The latter case is less likely, as these two USPs do not group closer to the green cluster which supposedly originated first. The existence of diverse domains in USP members in the yellow cluster signifies the functional diversity of USP members in plants. These proteins may have evolved to serve varied functions in protecting the plant from diverse stressors. Thus, it is imperative to accurately identify and further explore the diversity of these genes.

Molecular markers can be used as a selection tool for investigating genetic diversity, transferability of genes for genome editing purposes. In our study, we identified 19 SSR markers based on their high reproducibility, polymorphic genetic information, and hypervariable nature. As they produce high allelic variations among very closely related varieties, these markers can be used for the identification of specific *USP* members in future. In concordance with previous studies in Viridiplantae, we also found trinucleotide type as the dominant motif (Isokpehi et al., 2011).

Looking at the expression pattern of *OsUSP* genes we found that several members showed modulation under different abiotic stress conditions (Figure 6A). A number of these genes exhibited strong upregulation when exposed to these stressors. These findings fit well with previous reports of the stress-specific regulation of *USP* genes (Loukehaich et al., 2012; Jung et al., 2015; Melencion et al., 2017). Moreover, it has been demonstrated that overexpression of certain *USP* genes lead to enhanced tolerance to heat and osmotic stress. In relation to this, candidate *OsUSP* genes may also have similar functional roles in stress tolerance. On top of this, we found the presence of multiple stress-responsive regulatory elements upstream of *OsUSP* genes. These include – ABRE (ABA response), ARE (anoxia), LTR (low temperature), MBS (drought), TC-rich repeats (stress and defense), HSE (heat shock), etc. A previous report by Bhuria et al. (2016) showed that *AtUSP* promoter is highly inducible by multiple abiotic stressors and was able to produce multi-stress tolerance. In another study in cotton plants, the promoter of a *USP* gene was found to be responsive to dehydration, ABA, salt, heavy metals, and gibberellic acid (Zahur et al., 2009). Moreover, Wild tomato *USP* genes were induced under ABA, ethylene, drought, salt, heat, wounding, oxidative, and cold stress (Loukehaich et al., 2012). Similarly, the *SbUSP* gene from *Salicornia brachiata* also showed expression under salt, drought, cold, and heat stress (Udawat et al., 2014). Overall, these provide extensive evidence for the differential modulation of rice *USP* genes under abiotic stress conditions and that their promoters have the capacity to induce stress resilience, making them good candidates to drive multiple stress-responsive expression of transgenes in genetically engineered plants.

In coherence with this, during *in silico* expression profiling of *OsUSP* genes, we also found that expression of most rice *USP* genes was closely associated with heat, salinity, drought, dehydration, and submergence. Next, we used qRT-PCR to correlate the stress-specific function of the selected *USP* genes under multiple abiotic stresses. These led to the confirmation of the modulation of these rice *USP* genes under varying environmental stressors. For example, *OsUSP3* showed upregulation under all stress-conditions except cold which makes it a very suitable for downstream wet-lab validation (Figure 7). On the contrary, *OsUSP22* was mostly downregulated in the abiotic stressors except ABA and SA, indicating a strong modulation under stress. Interestingly, we observed that only *OsUSP3* and *OsUSP12* have exhibit elevated expression under oxidative stress. In addition, the response of each *USP* to the variety of stressors varied widely, indicating the possibility of diverse modes of transcriptional regulation for members of this gene family in rice. Uncovering these exact natures of such regulatory modules and their functional consequences can be of great importance for plant stress-oriented research.

To further bolster our understanding the rice *USP* gene family, we focused on analyzing the protein products of these genes. Proper functioning of proteins depends greatly on the post-translational modifications (PTMs) of proteins, glycosylation and phosphorylation being major two. In this regard, the glycosylation process plays a major role in protein folding, stability and signal transduction (Arey, 2012), whereas phosphorylation plays a critical role in the activation or deactivation of proteins by altering structural conformation (Cohen, 2002) and plays a significant role in the signaling pathways (Cutillas, 2017), metabolism and in combating various stress conditions (Zhang et al., 2020). In response to abiotic stresses in crop plants, PTM of proteins have been reported in response to abiotic stresses in crop plants and is suggested to be involved in various biological processes under natural conditions (Wu et al., 2016). Previous studies on protein PTM in soybean and rice under stresses showed that glycosylation occurs in response to flood and cold (Wu et al., 2016). Here, we identified 68 predicted glycosylation sites, among which 19 sites showed the highest chance of glycosylation. This confirms the ability of OsUSP members to perform glycosylation mediated secondary structure modification that may regulate the protein functions in stress condition. Furthermore, various studies suggested that different kinases are activated following that their activation loop residues are phosphorylated (Ravala et al., 2015) and autophosphorylation of UspA protein of *E. coli* also occur in response to stasis (Freestone et al., 1997). Moreover, phosphorylation mediated PTM was found prominent in combating multiple abiotic stressors in rice, maize, wheat, and chickpea (Wu et al., 2016). Thus, the predicted phosphorylation sites with position and specific kinases from the present work could be used for further confirmation of the role of phosphorylation in the function of different OsUSPs by using various *in vitro* techniques.

Moreover, a deep understanding of the binding affinity and binding site residues of inhibitors to OsUSPs is needed for the improvement of abiotic stress-resistant crops. In this present study, we have performed blind docking (Hetényi and van der Spoel, 2002) method for exploration of possible binding sites and binding affinity of our proteins and toward some known USP and kinase inhibitors. In concordance with the previous study on UspA inhibitor in *E. coli* (Bandyopadhyay et al., 2021), ZINC000104153710 showed the best docking result, thus suggesting having an inhibitory effect on OsUSP activity. Furthermore, studies have found flavonoids acting as an inhibitor toward kinases (Cassidy and Setzer, 2010). Here, Luteolin showed the best result when docked against kinase domain containing OsUSPs. This strongly suggests that Luteolin may hinder kinase activity upon binding. This will facilitate further simulation, investigation regarding the interaction of other inhibitors to these binding sites and identification of some novel inhibitors that may inhibit the activity of USP proteins.

## Conclusion

Overall, this study covers a comprehensive genome-wide scale investigation of *USP* gene family members in a staple crop, rice. In total, we were able to identify 44 *USP* genes in the monocot genome, a number slightly higher than previous reports. The key focus of this investigation was to perform an in-depth exploration of the structural and functional properties of the identified genes. This led us to discover the functional diversity of rice *USP* genes and their multi-stress regulatory nature. Moreover, using qRT-PCR, we were able validate this multi-stress responsive nature of promising *OsUSP* genes. However, further research is required to identify the functional contribution of these genes by generating overexpressing or knockout lines of *USP* genes alone or in combination. With the aid of modern biotechnological tools, the selected *OsUSP* genes could be targeted for improving not only rice but also other important crop species for developing multi-stress resilience plants.

## Data Availability Statement

The raw data supporting the conclusions of this article will be made available by the authors, without undue reservation.

## Author Contributions

TI conceived the idea and designed and supervised the experiments. SAr, AS, and SAk performed all the experiments and wrote the manuscript. TI and AS helped in data analysis. RS critically reviewed the manuscript. All authors read the manuscript and approved the final version.

## Conflict of Interest

The authors declare that the research was conducted in the absence of any commercial or financial relationships that could be construed as a potential conflict of interest.

## Publisher’s Note

All claims expressed in this article are solely those of the authors and do not necessarily represent those of their affiliated organizations, or those of the publisher, the editors and the reviewers. Any product that may be evaluated in this article, or claim that may be made by its manufacturer, is not guaranteed or endorsed by the publisher.

## Footnotes

1
http://rapdb.dna.affrc.go.jp/

2
http://pfam.sanger.ac.uk/

3
http://cello.life.nctu.edu.tw/

4
https://www.genscript.com/wolf-psort.html

5
http://www.cbs.dtu.dk/services/ChloroP/

6
https://plants.ensembl.org/info/data/ftp/index.html

7
http://viewer.shigen.info/oryzavw/maptool/MapTool.do

8
http://chibba.agtec.uga.edu/duplication/index/downloads

9
http://pgrc.ipk-gatersleben.de/misa/misa.html

10
http://www.cbs.dtu.dk/services/NetNGlyc/

11
https://services.healthtech.dtu.dk/service.php?NetPhos-3.1

12
https://genevestigator.com/gv/index.jsp

13
http://bioinformatics.psb.ugent.be/webtools/plantcare/html/

14
http://toolkit.tuebingen.mpg.de

15
http://www.pubchem.ncbi.nlm.nih.gov

## Supplementary Material

Supplementary Figure 1 Quality and quantification of isolated RNA.

Supplementary Figure 2 Homology modeling of seven OsUSP proteins having only USP domain.
