# Supplementary figures and images for: Comprehensive in silico Characterization of Universal Stress Proteins in Rice (Oryza sativa L.) With Insight Into Their Stress-Specific Transcriptional Modulation
Source: Front Plant Sci. 2021 Jul 28;12:712607. doi: 10.3389/fpls.2021.712607 (PMC8355530; doi:10.3389/fpls.2021.712607)

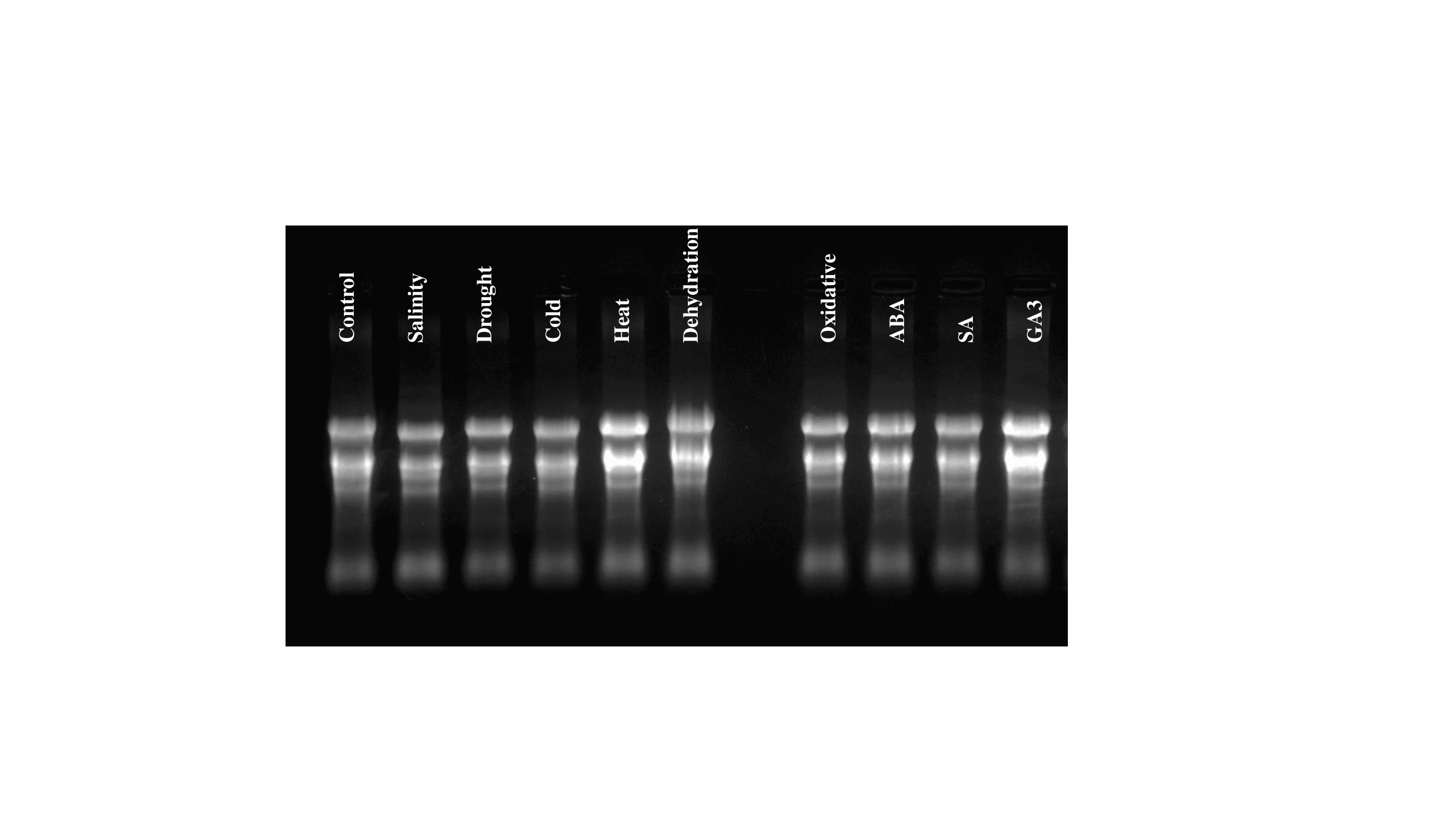

Supplement: Supplementary Figure 1 — Quality and quantification of isolated RNA. [file Image_1.TIFF]

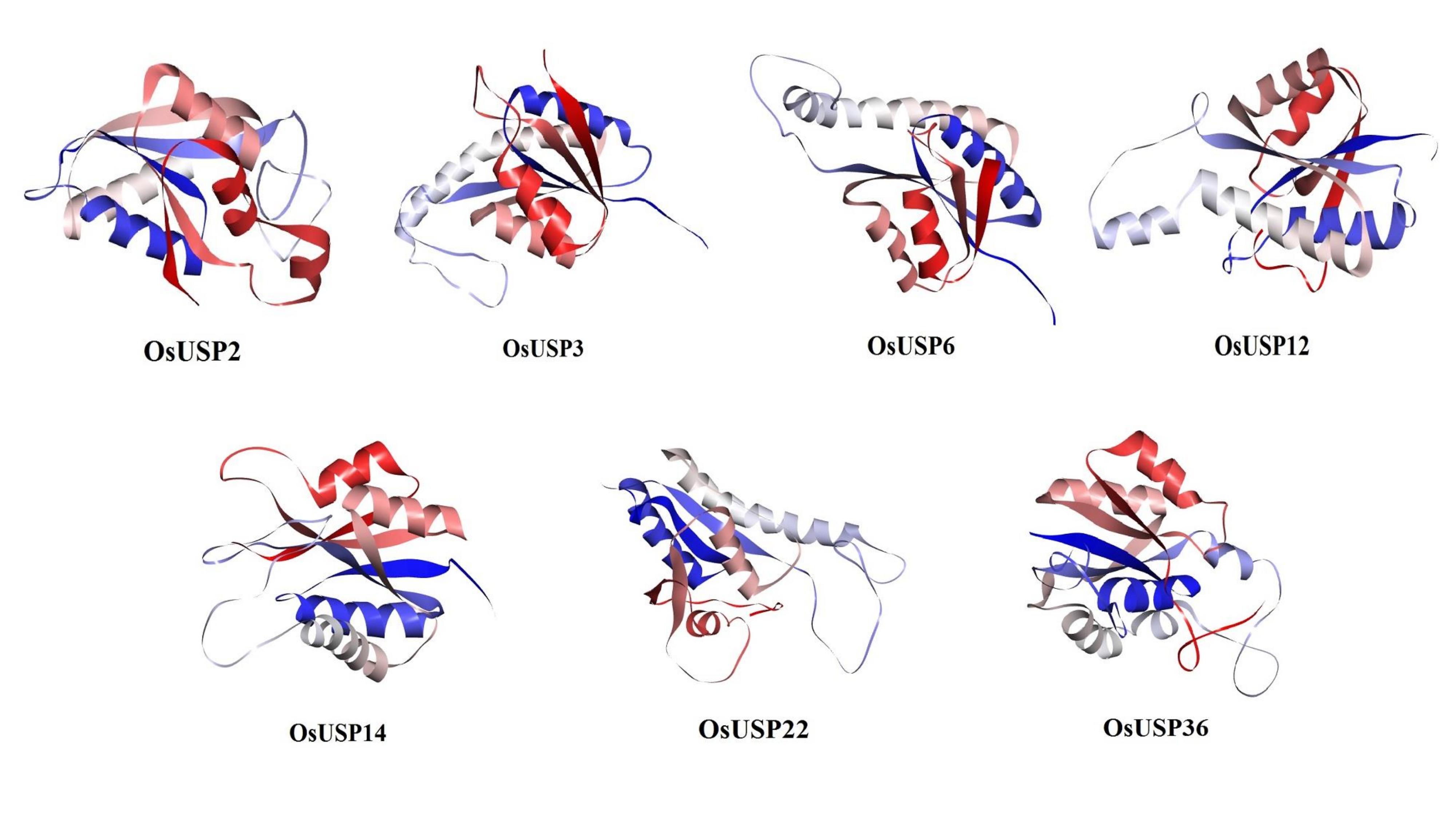

Supplement: Supplementary Figure 2 — Homology modeling of seven OsUSP proteins having only USP domain. [file Image_2.TIF]
