# Supplementary material for: Comprehensive in silico Characterization of Universal Stress Proteins in Rice (Oryza sativa L.) With Insight Into Their Stress-Specific Transcriptional Modulation
Source: Front Plant Sci. 2021 Jul 28;12:712607. doi: 10.3389/fpls.2021.712607 (PMC8355530; doi:10.3389/fpls.2021.712607)
Supplement: Supplementary file 7 [file Table_5.docx]

**Supplementary Table 5.** List of primers used for qRT-PCR.

| **Primer’s name** | **Sequence 5ʹ-3ʹ** |
| --- | --- |
| OsUSP2_FOR | GTGGAGACGATGGTGGAGAG |
| OsUSP2_REV | CAGTGATTGCTCACGCTTCC |
| OsUSP3_FOR | GGGGTGGCTGTAGAGACAAT |
| OsUSP3_REV | CTTTGTACAGGTCCACGGCT |
| OsUSP6_FOR | CCTTCGGACGAAGAGGTGTT |
| OsUSP6_REV | AGATTGAGCTCCCCCACAG |
| OsUSP12_FOR | GAAGTCATCTGCAGCGAGGT |
| OsUSP12_REV | GGACAGTCGGCATGCTTAAC |
| OsUSP14_FOR | AGGTATGGGATCAACCCCGA |
| OsUSP14_REV | GCCTCACAGAGTTTCTCCCT |
| OsUSP22_FOR | GATGATCCCGCTGGTGGAG |
| OsUSP22_REV | GAGCCAGTGCAGGGGAATC |
| OsUSP32_FOR | CTGCGAAAACGGGACACTTG |
| OsUSP32_REV | GGATGATCCGGTGCCTACAG |
| OsUSP33_FOR | GCTCTTGCTTGCACTAGGGA |
| OsUSP33_REV | ACGGCAACGACACTGATCTT |
| OsUSP36_FOR | ATGAAGGTGCTTGTGGCGG |
| OsUSP36_REV | GGCTCAATGGCATGGACAAG |
| eEF1_FOR | TTTCACTCTTGGTGTGAAGCAGAT |
| eEF1_REV | GACTTCCTTCACGATTTCATCGTAA |
