# Supplementary material for: Comprehensive in silico Characterization of Universal Stress Proteins in Rice (Oryza sativa L.) With Insight Into Their Stress-Specific Transcriptional Modulation
Source: Front Plant Sci. 2021 Jul 28;12:712607. doi: 10.3389/fpls.2021.712607 (PMC8355530; doi:10.3389/fpls.2021.712607)
Supplement: Supplementary file 8 [file Table_6.docx]

**Supplementary Table 6.** List of duplicated *OsUSP* genes with the probable dates of duplication

| 1. Sl. no | Locus 1 | Locus 2 | Ka | ks | Ka/ks | Duplication time (Mya) | Duplication type |
| --- | --- | --- | --- | --- | --- | --- | --- |
| 1 | *OsUSP1* | *OsUSP22* | 0.3313 | 1.9597 | 0.1680 | 150.77 | Segmental |
| 2 | *OsUSP2* | *OsUSP23* | 0.286 | 0.8168 | 0.3501 | 62.83 | Segmental |
| 3 | *OsUSP2* | *OsUSP17* | 0.3909 | 0.7172 | 0.5450 | 30.07 | Segmental |
| 4 | *OsUSP4* | *OsUSP29* | 0.7337 | 0 | - | - | Segmental |
| 5 | *OsUSP5* | *OsUSP26* | 0.1411 | 0.4644 | 0.3038 | 35.72 | Segmental |
| 6 | *OsUSP6* | *OsUSP25* | 0.3621 | 0.5189 | 0.6978 | 39.92 | Segmental |
| 7 | *OsUSP7* | *OsUSP24* | 0.1651 | 0.4417 | 0.3737 | 33.97 | Segmental |
| 8 | *OsUSP9* | *OsUSP29* | 0.8109 | 0 | - | - | Segmental |
| 9 | *OsUSP15* | *OsUSP28* | 0.1981 | 0.3718 | 0.5328 | 28.6 | Segmental |
| 10 | *OsUSP18* | *OsUSP31* | 0.4294 | 0.4148 | 1.035 | 31.91 | Segmental |
| 11 | *OsUSP39* | *OsUSP40* | 0.3247 | 1.4484 | 0.2241 | 111.41 | Tandem |
