# Supplementary material for: Comprehensive in silico Characterization of Universal Stress Proteins in Rice (Oryza sativa L.) With Insight Into Their Stress-Specific Transcriptional Modulation
Source: Front Plant Sci. 2021 Jul 28;12:712607. doi: 10.3389/fpls.2021.712607 (PMC8355530; doi:10.3389/fpls.2021.712607)
Supplement: Supplementary file 9 [file Table_7.docx]

**Supplementary Table 7.** Identification of Microsatellite Markers in *OsUSPs.*

| **Type** | **SSR Motif** | **Number** | **Frequency** |
| --- | --- | --- | --- |
| **Mononucleotide** | T | 1 | 5.26 |
| **Dinucleotide** | CG | 1 | 5.26 |
| **Trinucleotide** | AGC | 1 | 89.47 |
|  | AGG | 1 |  |
|  | CCG | 1 |  |
|  | CGC | 1 |  |
|  | CGG | 1 |  |
|  | CGT | 1 |  |
|  | GCG | 9 |  |
|  | GGA | 1 |  |
|  | GGC | 1 |  |
