# Supplementary material for: Comprehensive in silico Characterization of Universal Stress Proteins in Rice (Oryza sativa L.) With Insight Into Their Stress-Specific Transcriptional Modulation
Source: Front Plant Sci. 2021 Jul 28;12:712607. doi: 10.3389/fpls.2021.712607 (PMC8355530; doi:10.3389/fpls.2021.712607)
Supplement: Supplementary file 10 [file Table_8.docx]

**Supplementary Table 8.** Detailed information about predicted glycosylation sites in OsUSP proteins.

| Protein | Position | Region | Score | Protein | Position | Region | Score |
| --- | --- | --- | --- | --- | --- | --- | --- |
| OsUSP5 | 4 | NPSS | 0.7624 | OsUSP28 | 609 | NVTQ | 0.7967 |
| OsUSP8 | 231 | NISE | 0.7193 | OsUSP32 | 144 | NCSV | 0.7081 |
| OsUSP8 | 269 | NLSE | 0.6451 | OsUSP32 | 379 | NGTL | 0.7171 |
| OsUSP10 | 108 | NATV | 0.7112 | OsUSP33 | 585 | NGSL | 0.6798 |
| OsUSP10 | 844 | NLSV | 0.6419 | OsUSP34 | 130 | NVSM | 0.6528 |
| OsUSP15 | 183 | NVST | 0.7482 | OsUSP38 | 217 | NLTW | 0.7513 |
| OsUSP23 | 3 | NVTG | 0.7383 | OsUSP40 | 238 | NIST | 0.7127 |
| OsUSP27 | 29 | NGSR | 0.7749 | OsUSP41 | 241 | NIST | 0.7192 |
| OsUSP27 | 423 | NKTG | o.6938 | OsUSP41 | 503 | NLTW | 0.6106 |
| OsUSP27 | 527 | NGSL | 0.6572 |  |  |  |  |
