# Supplementary material for: Comprehensive in silico Characterization of Universal Stress Proteins in Rice (Oryza sativa L.) With Insight Into Their Stress-Specific Transcriptional Modulation
Source: Front Plant Sci. 2021 Jul 28;12:712607. doi: 10.3389/fpls.2021.712607 (PMC8355530; doi:10.3389/fpls.2021.712607)
Supplement: Supplementary file 11 [file Table_9.docx]

**Supplementary Table 9.** Detailed information about predicted phosphorylation sites in OsUSP proteins.

| **Protein** | **Number of sites** | **Protein** | **Number of sites** |
| --- | --- | --- | --- |
| OsUSP1 | 19 | OsUSP23 | 8 |
| OsUSP2 | 8 | OsUSP24 | 13 |
| OsUSP3 | 3 | OsUSP25 | 3 |
| OsUSP4 | 52 | OsUSP26 | 13 |
| OsUSP5 | 11 | OsUSP27 | 57 |
| OsUSP6 | 7 | OsUSP28 | 45 |
| OsUSP7 | 13 | OsUSP29 | 61 |
| OsUSP8 | 34 | OsUSP30 | 13 |
| OsUSP9 | 45 | OsUSP31 | 8 |
| OsUSP10 | 45 | OsUSP32 | 19 |
| OsUSP11 | 5 | OsUSP33 | 48 |
| OsUSP12 | 6 | OsUSP34 | 41 |
| OsUSP13 | 1 | OsUSP35 | 42 |
| OsUSP14 | 5 | OsUSP36 | 6 |
| OsUSP15 | 45 | OsUSP37 | 9 |
| OsUSP16 | 33 | OsUSP38 | 35 |
| OsUSP17 | 10 | OsUSP39 | 47 |
| OsUSP18 | 4 | OsUSP40 | 47 |
| OsUSP19 | 9 | OsUSP41 | 53 |
| OsUSP20 | 83 | OsUSP42 | 13 |
| OsUSP21 | 4 | OsUSP43 | 5 |
| OsUSP22 | 7 | OsUSP44 | 9 |
